# Factors influencing HIV testing and counselling services among men who have sex with men in Western China: a cross-sectional study based on Andersen’s Behavioral Model

**DOI:** 10.1265/ehpm.22-00066

**Published:** 2022-05-27

**Authors:** Bing Lin, Jiaxiu Liu, Yingjie Ma, Xiaoni Zhong

**Affiliations:** 1 School of Public Health and Management, Research Center for Medicine and Social Development, Chongqing Medical University, Chongqing, China; 2 School of Medical Informatics, Chongqing Medical University, Chongqing, China

**Keywords:** HIV testing, HIV counselling, Health service, Men who have sex with men, Andersen’s Behavioral Model

## Abstract

**Background:**

Men who have sex with men (MSM), as a marginal population, has been largely ignored by health service projects. We assessed the utilization of HIV testing and counselling services and its influencing factors based on Andersen’s Behavioral Model, so as to provide a theoretical basis for future infectious disease prevention and control strategies and health services policy formulation for these population.

**Method:**

This was a cross-sectional study. A sample survey was conducted in Western China, and an anonymous self-administered questionnaire survey was conducted among MSM. Based on Andersen’s Behavioral Model, the questionnaire divided the influencing factors into predisposing factor, enabling factor and need factor. Multivariate logistic regression analysis was used to explore the factors influencing the utilization of HIV testing and counselling.

**Results:**

There were 3184 valid questionnaires. In the survey of HIV health services, 82.85% MSM had HIV testing and 64.98% MSM had HIV counselling, respectively. Among the predisposing factor, age 25 years old and over was a facilitator of HIV testing and counselling, and ethnicity was a factor associated with HIV testing. Among the enabling factor, MSM living in urban were more likely to have access to testing and counselling services, and income was also linked to HIV testing. Among the need factor, a high level of HIV knowledge could promote testing and counselling, and a history of sexually transmitted diseases (STD) was a facilitator of testing.

**Conclusions:**

HIV testing is widespread in Western China and higher than counselling service. MSM with high-risk characteristics should be identified as a priority in the future public health services.

## Introduction

As a chronic infectious disease, acquired immune deficiency syndrome (AIDS) has seriously affected the development of global public health undertakings. The third Sustainable Development Goal (SDG-3) includes a target, which is end the epidemic of HIV/AIDS by 2030 (Project 2030) [1]. Until now, the global decline in AIDS-related deaths is on track, while the decline in new HIV infections is off track to achieve the Project 2030 [2]. The feasibility of achieving the Project 2030 is not ideal.

Moreover, AIDS epidemic situation in China is also not optimistic. In recent years, the AIDS epidemic in China has grown rapidly, from 440,000 in 2011 to 950,000 in 2019 [3]. Homosexual transmission has become the second most common HIV transmission route after heterosexual transmission, accounting for about a quarter of new HIV cases [4]. Men who have sex with men (MSM) is the main key population of human immunodeficiency virus (HIV) infection [3]. Epidemiological evidence suggested that prevention programs for MSM are a top priority response to AIDS in China [5]. MSM has not been widely accepted by the society in China. Bound by traditional moral concepts and under the double pressure of the society and family, many MSM choose to marry with the opposite sex and hide their sexual orientation. However, many MSM still choose to have sex with male partners after marriage, so as to expose their heterosexual spouses or sexual partners to a high-risk environment for HIV, which then spread to the general population. Despite some success in HIV prevention efforts, the rate of HIV transmission among MSM population still remains high [6]. Chinese MSM are increasingly at risk of HIV infection, with the proportion of new HIV infections increasing from 2.5% to 25.5%, despite various interventions were adopted [7, 8]. Therefore, it is important to focus on MSM population for the prevention and control of AIDS.

Health services mainly refer to medical treatment, prevention, health care, rehabilitation and other activities provided to residents by the health system with certain health resources, including prevention and control of infectious diseases, disease screening [9] and disease surveillance [10]. HIV-related health services mainly include HIV testing and HIV counselling. Existing countries have recognized the importance of strengthening public health services to combat infectious diseases and conducted continuous research and improvement. A Canadian study found that the private health services of HIV counselling and point-of-care (POC) testing based on urban communities were acceptable and feasible to a certain extent [11]. In addition, the Internet can be used as a potential point of access to health screening to address inequalities in health services, according to an US online survey [12]. And some Chinese scholars also have integrated the newly developed mobile health (mHealth) intervention with HIV health services (For example, weekly reminders were sent to participants via text messages, and articles about self-management of AIDS were sent three times a week though social media app) to prevent disease transmission and reduce HIV infection rate [13].

However, in the study on health services, the priority groups often focus on: adolescents [14], mothers and children [15], migrants [16] or female sex workers [17]. MSM, as a special population, has been largely ignored by health service projects [18]. In areas with laws and policies forbidden homosexuality, these high-risk key population is more difficult to access [14]. Meanwhile, MSM also face many obstacles in their participation in health services. There is an evidence that MSM have been rejected by family members, publicly humiliated and laughed by health care workers when they disclose their sexual orientation [19]. Because of the stigma and discrimination from health providers and neglect by health systems, it poses a significant challenge to HIV care and service for this population. As a result, more attentions have to be paid to the need and utilization of health services for MSM population, which is beneficial to understand and address the complex situation of limited MSM access to HIV health services.

Reasonable evaluation of acquisition and quality of health services can help reduce disparities and health care inequalities in MSM and other sexual minorities populations [20]. Our study assumes that there are objective and subjective factors potentially affecting the HIV testing and counselling service behaviors of MSM population, which requires a theoretical model as support and guidance. Therefore, our study takes HIV testing and HIV counselling as health services utilization items, and discusses the utilization and influencing factors of health services among MSM population based on Andersen’s Behavioral Model. The model was originally developed to describe the factors that influence the use of health services, and it divided the influencing factors into predisposing factor, enabling factor and need factor [21]. The various factors in the model represent different characteristics. Predisposing factor refers to an individual’s unchangeable nature, including demographic variables and social structural variables. Enabling factor refers to that can promote or hinder the use of services, including personal resources and regional health services resources. Need factor refers to the subjective understanding of the disease and clinical objective diagnosis results [22]. It has been widely used to guide the examination of predictors associated with a variety of health outcomes, such as drug use among people living with HIV [23], hypertension disease management [12], women’s mental health [22], breast cancer screening [24, 25]. Assessing health care inequities in sexual minorities, including barriers to health care access, utilization rates, and the quality of care received, is a priority research area and may help reduce disparities in MSM population [12]. Taking Andersen’s Behavioral Model as the theoretical basis and incorporating it into the analysis of influencing factors can better explain and understand the influence of variables on outcome indicators, and can also better clarify the context of health services, so as to provide targeted suggestions for future policy making for MSM population. In this study, we (1) investigate the health service utilization of HIV testing and HIV counselling, and (2) examine the associated factors for HIV testing and counselling separately for MSM.

## Method

### Participants and procedures

This was a cross-sectional study derived from two phases: 2013 to 2014 and 2019 to 2021 respectively, and accompanied by two five-year national key project for infectious diseases of the Ministry of Science and Technology of China. MSM refers to men who have sex with men regardless of their self-identified sexual orientation (homosexual, heterosexual or bisexual). Qualified participants in Western China (Chongqing, Xinjiang, Sichuan and Guangxi) were found through collaboration with local non-governmental organizations (NGOs) and peer recommendation. Inclusion criteria included: (1) physiological male (assigned male sex at birth), (2) aged 18∼65 years old, (3) had engaged in sex with male partners in the past 6 months, (4) willing to participate and provided informed. The method of anonymous self-filling questionnaire was adopted, which was collected on the spot and checked for completeness and logic.

### Definitions of HIV testing and counselling

HIV health services in this study include HIV testing and HIV counselling. HIV testing refers to whether the participants had been tested for HIV, and they will choose “yes” or “no”. HIV counselling refers to whether the participants have had AIDS-related counselling, and they will choose “yes” or “no”.

### Factors of Andersen’s Behavioral Model

**Fig. 1:**
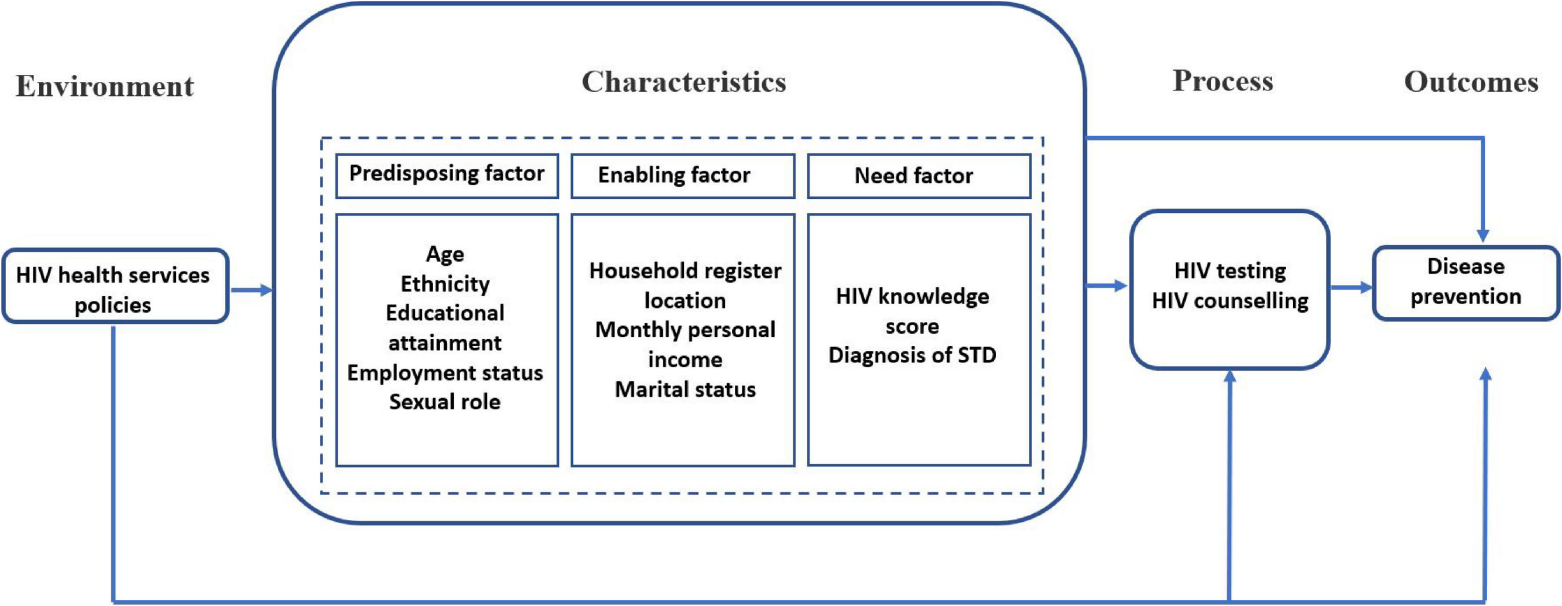
A diagram of the theoretical framework of Andersen’s Behavioral Model. HIV: human immunodeficiency virus. STD: sexually transmitted disease.

The model divided the influencing factors into: predisposing factor, enabling factor and need factor. Predisposing factor is mainly represented by demographic characteristics and social structure characteristics that might help explain limited access to care. Age, ethnicity, educational attainment and employment status are included into this factor. Sexual role is the way of having penetrative sex with a male partner, such as the inserter (like “1”) and the receiver (like “0”). The predisposing factor in Andersen’s Behavioral Model can also refer to the susceptibility factors that distinguish traditionally vulnerable groups with respect to access to medical care. For example, current study have classified sexual orientation as predisposing factor [12]. Therefore, we believe that sexual role can also be attributed to this factor. Enabling factor indicate factors that might facilitate or impede access to care. Household register location, monthly personal income and marital status are included into this factor. Monthly personal income was calculated in RMB, the legal tender of China, and the unit is Yuan. Need factors expected to have a substantial bearing on access to care. HIV knowledge score and diagnosis of sexually transmitted disease (STD) are included into this factor. The HIV knowledge scale (Cronbach’s alpha = 0.672) was made up of 13 questions based on a revision of the International AIDS Knowledge Survey General Scale [26]. The answers include “true”, “false” and “don’t know”. Based on the answers, there is a 1 point for correct answers, and 0 points for incorrect or unknown answers. The higher score is, the more knowledge the respondents had about HIV. We consider that the score ⩾11 indicates a good level of HIV knowledge [8, 27]. A diagram of the theoretical framework of Andersen’s Behavioral Model is shown in Fig. 1.

### Statistical analysis

The variables in the questionnaire were classified and described, and then univariate differences were analyzed by Chi-square test according to the outcome variables of health services: HIV testing and HIV counselling. Since the data analyzed were from the two phases of the survey, we also classified the different time stages. The multivariate logistic regression models of HIV testing and HIV counselling were established respectively, and adjusted the time phase factor of the survey. To explore the influence of the three factors in Andersen’s Behavioral Model, all variables were included and screened using stepwise regression in multivariate logistic regression analysis. Stepwise regression adopted backward selection method, with slentry = 0.05 as the criterion for variable inclusion and slstay = 0.05 as the criterion for variable exclusion. The p-value < 0.05 was considered statistically significant. All statistical analyses were performed using SAS V.9.4 software.

## Results

**Fig. 2:**
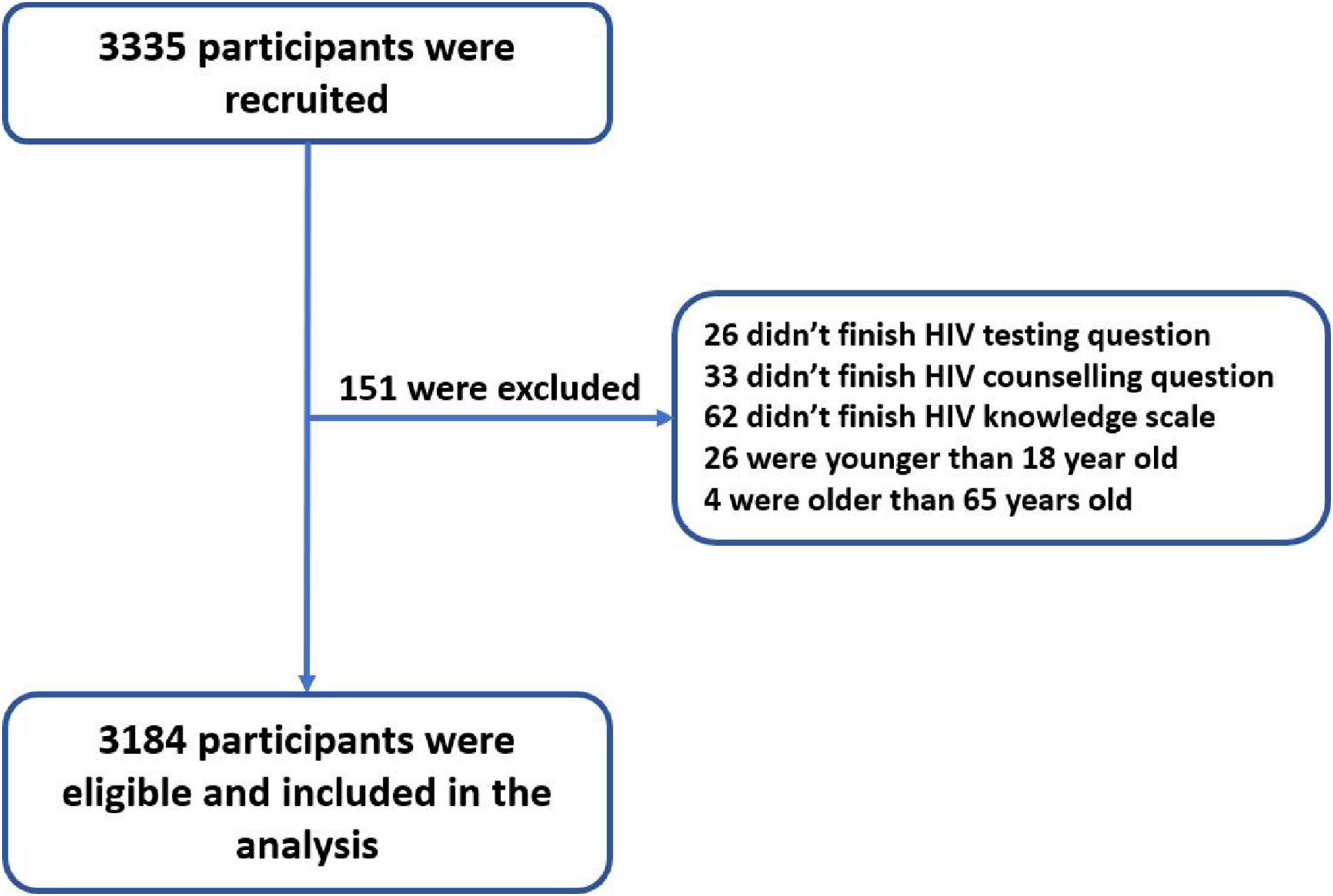
Flow chart of participants’ enrolments. HIV: human immunodeficiency virus.

A total of 3335 MSM were recruited for our survey. 3184 valid questionnaires were collected from Western China in this study, and the effective recovery rate was 95.47%. A total of 151 MSM were excluded for the reasons shown in Fig. 2. 1726 (54.21%) MSM were surveyed from 2013 to 2014 and 1458 (45.79%) MSM were surveyed from 2019 to 2021. 2638 (82.85%) MSM had HIV testing and 2069 (64.98%) MSM had HIV counselling, respectively.

Age, ethnicity, educational attainment, employment status, sexual role, household register location, monthly personal income and HIV knowledge score were statistically different in HIV testing rates among the MSM population (all p-values < 0.05). Age, educational attainment, employment status, household register location, monthly personal income and HIV knowledge score were statistically different in HIV counselling rates (all p-values < 0.05). Meanwhile, HIV testing rates (88.61% vs. 77.98%, p < 0.001) and HIV counselling rates (70.10% vs. 60.66%, p < 0.001) were significantly higher in the phase 2 (2019∼2021) compared to phase 1 (2013∼2014) (Table 1 and Table 2).

**Table 1:** Univariate analysis of HIV testing service (N = 3184)

**Items**	**All**	**Yes group of HIV testing** **(N = 2638)** **N (%)**	**No group of HIV testing** **(N = 546)** **N (%)**	**p-value**
***Predisposing factor***				
Age				
18∼25 years old	354	261 (9.90)	93 (17.03)	**<0.001^^^**
25∼35 years old	1501	1228 (46.59)	273 (50.00)	
≥35 years old	1327	1147 (43.51)	180 (32.97)	
Missing data	2	2	0	
Ethnicity				
Ethnic Han	2936	2421 (91.84)	515 (94.32)	**0.048^^^**
Ethnic minorities	246	215 (8.16)	31 (5.68)	
Missing data	2	2	0	
Educational attainment				
Junior high or below	366	271 (10.29)	95 (17.40)	**<0.001^^^**
Senior high	775	619 (23.51)	156 (28.57)	
Junior college	775	652 (24.76)	123 (22.53)	
College or above	1263	1091 (41.44)	172 (31.50)	
Missing data	5	5	0	
Employment status				
Employed	2457	2101 (79.85)	356 (65.56)	**<0.001^^^**
Retirement	39	30 (1.14)	9 (1.66)	
Student	398	283 (10.76)	115 (21.18)	
Unemployed	280	217 (8.25)	63 (11.60)	
Missing data	10	7	3	
Sexual role*				
Only “1”	908	763 (29.39)	145 (27.00)	**0.004^^^**
Both, mainly “1”	611	524 (20.19)	87 (16.20)	
Both of it	755	617 (23.77)	138 (25.70)	
Both, mainly “0”	456	382 (14.71)	74 (13.78)	
Only “0”	403	310 (11.94)	93 (17.32)	
Missing data	51	42	9	
***Enabling factor***				
Household register location				
Urban	2205	1887 (71.89)	318 (58.35)	**<0.001^^^**
Rural	965	738 (28.11)	227 (41.65)	
Missing data	14	13	1	
Monthly personal income				
≤1000 RMB	411	291 (11.11)	120 (22.30)	**<0.001^^^**
1000∼3000 RMB	906	719 (27.46)	187 (34.76)	
3000∼5000 RMB	1062	901 (34.42)	161 (29.93)	
>5000 RMB	777	707 (27.01)	70 (13.01)	
Missing data	28	20	8	
Marital status				
Unmarried	2397	1989 (75.51)	408 (75.00)	0.435^^^
Married	512	415 (15.76)	97 (17.83)	
Divorced	268	230 (8.73)	39 (7.17)	
Missing data	6	4	2	
***Need factor***				
HIV knowledge score				
≥11	1320	1221 (46.29)	99 (18.13)	**<0.001^^^**
<11	1864	1417 (53.71)	447 (81.87)	
Missing data	0	0	0	
Diagnosis of STD				
Yes	228	198 (7.53)	30 (5.54)	0.101^^^
No	2942	2430 (92.47)	512 (94.46)	
Missing data	14	10	4	
Phase of the study				
Phase 1 (2013∼2014)	1726	1346 (51.02)	380 (69.60)	**<0.001^^^**
Phase 2 (2019∼2021)	1458	1292 (48.98)	166 (30.40)	
Missing data	0	0	0	

*: Sexual role is the way of having penetrative sex with a male partner, such as the inserter (like “1”) and the receiver (like “0”).

^: The statistical method used was the Chi-square test. Bold fonts represent statistical differences.

HIV: human immunodeficiency virus. MSM: men who have sex with men. STD: sexually transmitted disease. RMB: the legal tender of China, the unit is Yuan.

**Table 2:** Univariate analysis of HIV counselling service (N = 3184)

**Items**	**All**	**Yes group of HIV counselling** **(N = 2069)** **N (%)**	**No group of HIV counselling** **(N = 1115)** **N (%)**	**p-value**
***Predisposing factor***				
Age				
18∼25 years old	354	200 (9.67)	154 (13.82)	**<0.001^^^**
25∼35 years old	1501	938 (45.36)	563 (50.54)	
≥35 years old	1327	930 (44.97)	397 (35.64)	
Missing data	2	1	1	
Ethnicity				
Ethnic Han	2936	1906 (92.21)	1030 (92.38)	0.867^^^
Ethnic minorities	246	161 (7.79)	85 (7.62)	
Missing data	2	2	0	
Educational attainment				
Junior high or below	366	207 (10.02)	159 (14.28)	**0.001^^^**
Senior high	775	493 (23.86)	282 (25.34)	
Junior college	775	526 (25.46)	249 (22.37)	
College or above	1263	840 (40.66)	423 (38.01)	
Missing data	5	3	2	
Employment status				
Employed	2457	1640 (79.46)	817 (73.60)	**<0.001^^^**
Retirement	39	25 (1.21)	14 (1.26)	
Student	398	226 (10.95)	172 (15.50)	
Unemployed	280	173 (8.38)	107 (9.64)	
Missing data	10	5	5	
Sexual role*				
Only “1”	908	606 (29.59)	302 (27.83)	0.351^^^
Both, mainly “1”	611	406 (19.82)	205 (18.89)	
Both of it	755	498 (24.32)	257 (23.69)	
Both, mainly “0”	456	290 (14.16)	166 (15.30)	
Only “0”	403	248 (12.11)	155 (14.29)	
Missing data	51	21	30	
***Enabling factor***				
Household register location				
Urban	2205	1497 (72.63)	708 (63.84)	**<0.001^^^**
Rural	965	564 (27.37)	401 (36.16)	
Missing data	14	8	6	
Monthly personal income				
≤1000 RMB	411	238 (11.57)	173 (15.73)	**<0.001^^^**
1000∼3000 RMB	906	554 (26.95)	352 (32.00)	
3000∼5000 RMB	1062	726 (35.31)	336 (30.54)	
>5000 RMB	777	538 (26.17)	239 (21.73)	
Missing data	28	13	15	
Marital status				
Unmarried	2397	1550 (75.02)	847 (76.17)	0.137^^^
Married	512	327 (15.83)	185 (16.64)	
Divorced	269	189 (9.15)	80 (7.19)	
Missing data	6	3	3	
***Need factor***				
HIV knowledge score				
≥11	1320	1009 (48.77)	311 (27.89)	**<0.001^^^**
<11	1864	1060 (51.23)	804 (72.11)	
Missing data	0	0	0	
Diagnosis of STD				
Yes	228	159 (7.71)	69 (6.23)	0.126^^^
No	2942	1904 (92.29)	1038 (93.77)	
Missing data	14	6	8	
Phase of the study				
Phase 1 (2013∼2014)	1726	1047 (50.60)	679 (60.90)	**<0.001^^^**
Phase 2 (2019∼2021)	1458	1022 (49.40)	436 (39.10)	
Missing data	0	0	0	

*: Sexual role is the way of having penetrative sex with a male partner, such as the inserter (like “1”) and the receiver (like “0”).

^: The statistical method used was the Chi-square test. Bold fonts represent statistical differences.

HIV: human immunodeficiency virus. MSM: men who have sex with men. STD: sexually transmitted disease. RMB: the legal tender of China, the unit is Yuan.

According to logistic regression analysis, among the predisposing factor, age 25 years old and over was a positive factor to HIV testing in the MSM population (25∼35 years old: OR = 2.18, 95%CI: 1.54∼3.09, p = 0.048; ⩾35 years old: OR = 3.00, 95%CI: 2.07∼4.35, p < 0.001). MSM of ethnic Han have significantly lower HIV testing rates compared to ethnic minorities (OR = 0.60, 95%CI: 0.39∼0.94, p = 0.026). Among the enabling factor, living in urban area (OR = 1.47, 95%CI: 1.18∼1.82, p < 0.001) and monthly personal income of >5000 RMB (OR = 2.24, 95%CI: 1.54∼3.26, p < 0.001) were positive factors to HIV testing. Among the need factor, a high level of HIV knowledge (OR = 3.58, 95%CI: 2.79∼4.58, p < 0.001) and a history of diagnosis with STD (OR = 1.61, 95%CI: 1.05∼2.49, p = 0.031) were positive factors to HIV testing (Table 3).

**Table 3:** Multivariate logistic regression analysis of HIV testing among MSM population based on Andersen’s Behavioral Model

**Variables**	**OR**	**95%CI**	**p-value**
***Predisposing factor***			
Age			
18∼25 years old	Reference	-	-
25∼35 years old	2.18	1.54∼3.09	**0.048^^^**
≥35 years old	3.00	2.07∼4.35	**<0.001^^^**
Ethnicity			
Ethnic Han	0.60	0.39∼0.94	**0.026^^^**
Ethnic minorities	Reference	-	-
***Enabling factor***			
Household register location			
Urban	1.47	1.18∼1.82	**<0.001^^^**
Rural	Reference	-	-
Monthly personal income			
≤1000 RMB	Reference	-	-
1000∼3000 RMB	1.41	1.05∼1.89	0.400^^^
3000∼5000 RMB	1.67	1.23∼2.23	0.275^^^
>5000 RMB	2.24	1.54∼3.26	**<0.001^^^**
***Need factor***			
HIV knowledge score			
≥11	3.58	2.79∼4.58	**<0.001^^^**
<11	Reference	-	-
Diagnosis of STD			
Yes	1.61	1.05∼2.49	**0.031^^^**
No	Reference	-	-

^: The statistical method used was the multivariate stepwise logistic regression. Stepwise regression adopted backward selection method, with slentry = 0.05 as the criterion for variable inclusion and slstay = 0.05 as the criterion for variable exclusion. Bold fonts represent statistical differences.

HIV: human immunodeficiency virus. MSM: men who have sex with men. STD: sexually transmitted disease. OR: odds ratio. CI: confidential interval. RMB: the legal tender of China, the unit is Yuan.

For HIV counselling, age of ⩾35 years old in the predisposing factor (OR = 2.15, 95%CI: 1.61∼2.86, p < 0.001), urban area in the enabling factor (OR = 1.27, 95%CI: 1.07∼1.51, p = 0.007), and high HIV knowledge in the need factor (OR = 2.19, 95%CI: 1.85∼2.59, p < 0.001) were influencing factors that facilitated counselling in the MSM population (Table 4).

**Table 4:** Multivariate logistic regression analysis of HIV counselling among MSM population based on Andersen’s Behavioral Model

**Variables**	**OR**	**95%CI**	**p-value**
***Predisposing factor***			
Age			
18∼25 years old	Reference	-	-
25∼35 years old	1.42	1.08∼1.87	0.730^^^
≥35 years old	2.15	1.61∼2.86	**<.001^^^**
***Enabling factor***			
Household register location			
Urban	1.27	1.07∼1.51	**0.007^^^**
Rural	Reference	-	-
***Need factor***			
HIV knowledge score			
≥11	2.19	1.85∼2.59	**<.001^^^**
<11	Reference	-	-

^: The statistical method used was the multivariate stepwise logistic regression. Stepwise regression adopted backward selection method, with slentry = 0.05 as the criterion for variable inclusion and slstay = 0.05 as the criterion for variable exclusion. Bold fonts represent statistical differences.

HIV: human immunodeficiency virus. MSM: men who have sex with men. STD: sexually transmitted disease. OR: odds ratio. CI: confidential interval.

## Discussion

Among the health services utilization projects examined in this study, the utilization of HIV testing (82.85%) was good, and the counselling services (64.98%) was significantly lower. HIV testing is a key strategic tool for HIV prevention for high-risk population. Most infected populations are not receiving effective antiretroviral therapy due to lack of widespread HIV testing [28]. In addition, it has also been suggested that frequent HIV testing in MSM can reduce health sector expenditures [29]. However, several studies have shown that HIV testing among MSM is not as satisfactory as expected [30, 31]. Based on the findings of HIV testing in Tianjin [32] and Ningbo [33], the testing rates were 69.95% (sample size = 4316) and 57.1% (sample size = 988), respectively, which were lower than our results. We have reason to believe that HIV testing is better among the MSM population in Western China. In addition, HIV counselling is also important for HIV prevention. A previous study showed that promoting regular counselling among MSM in Kenya helped reduce risky sexual behavior [34]. However, our results showed a relatively low utilization of HIV counselling in the MSM population and a mismatch with HIV testing services. This suggests the need to strengthen HIV counselling services. Only a simultaneous increase in the utilization of HIV testing and HIV counselling services will be effective in minimizing the risk of HIV infection in the MSM population.

At the same time, in both phases of the survey, we also found that the HIV testing and counselling rates increased significantly, probably due to the effect of the publicity in the past few years. For example, according to the performance report of the National Health Commission, remarkable progress has been made in the prevention and control of HIV/AIDS. The detection of infected persons has been intensified, with the number of people tested rising from 100 million in 2012 to 200 million in 2017 [35]. The government actively encouraged and supported social organizations to participate in prevention and control work, and also introduced a policy of “Four Frees and One Care”, which included free for HIV testing and counselling. Until now, the Chinese government has issued a series of HIV/AIDS prevention and control policies, regulations and guidelines, established a working mechanism under the leadership of government organizations and the participation of the whole society, and explored effective strategies or measures suitable for national conditions. Therefore, it may show that the efforts made in HIV/AIDS prevention and control have increased the HIV health services of MSM to a certain extent.

Andersen’s Behavioral Model was adapted to identify factors associated with HIV health services by including sets of variables. The model may help uncover factors ignored before, especially among MSM. According to the model, need factor reflects how people view their own health, subjective cognition of disease and clinical diagnosis for individual physical condition, is the most direct and important factor which influences health services utilization. It is considered to be one of the powerful predictor in health services [36]. It can also be seen from this study that need factor is the main factor affecting HIV testing and counselling services. Among them, HIV knowledge had statistically significant effects.

In the 3184 valid questionnaires collected this time, the average HIV knowledge score was 9.53 ± 2.55, among which, ≥11 accounted for 41.46%, indicating that the degree of HIV knowledge was generally moderate. HIV knowledge is the main factor affecting HIV testing and counselling, which is also consistent with the research ideas of Sofia [37]. In fact, as early as 10 years ago, scholars proposed the Information, Motivation and Behavioral Skills (IMB) Model, which has guiding significance in HIV risk reduction interventions [38]. The information in the IMB model mainly includes subjective information and objective information, of which objective information includes knowledge [39]. More and more studies have found that HIV knowledge plays a key role in the prevention and control of AIDS. The studies of Simukai and Doris [40, 41] also indicated that HIV knowledge and attitude were associated with non-condom use and high-risk sexual behavior, so knowledge promotion and popularization based on media platforms were urgently needed. At the same time, Chilot [42] has pointed out that a very low comprehensive knowledge of HIV/AIDS is one of the major reasons for the increase of HIV infections. It has been confirmed in the literature that educational programs [43], sexual education and communication activities [44] can contribute to the improvement of knowledge. It provides more ideas for improving knowledge and promoting HIV testing and counselling services among MSM population. We recommend more public health campaigns in local communities and more HIV-related knowledge on social networking platforms.

The enabling factor refers to the resources or means by which an individual has access to health services, usually involving individual and community resources such as health insurance, income, wealth, availability of services and urban classes. User fees for healthcare services present a barrier to patients accessing healthcare and reduce detection of serious infectious diseases [45]. In China, HIV testing and counselling is free only in Centers for Disease Control (CDC) or voluntary non-governmental organizations (NGOs), and in other institutions such as hospitals is charged. They are also not covered by medical insurance. At the same time, the location of HIV testing and counselling is only under some settings, which makes it easier for MSM living in urban areas to access health services. As a part of enabling factor, it reflects more realistic social problems in health services. It is therefore proposed to increase the number of health service points in rural areas in order to address the uneven distribution of resources between regions.

The predisposing factor refers to the nature of the individual that cannot be easily changed. Our study found that MSM with age (18∼25 years old) was less likely to test and counselling about HIV. This result differs from the results of foreign studies. Men in this age group (20∼24 years old) were more likely to be tested [31]. Previous studies have reported similar results [46]. However, findings in China have shown that MSM younger than 24 years old are associated with behaviors that impede HIV testing [32]. This is consistent with our results. We believe that young MSM (under 25 years old) are the primary target group for HIV testing and counselling. HIV prevalence among young MSM in China has been on the rise [47]. Young MSM may have a stronger sexual desire and thus engage in high-risk sexual behavior. However, lack of knowledge about HIV/AIDS and fear of a positive result may discourage young MSM from getting tested for HIV [48]. Therefore, it is important to focus on HIV health services utilization among the young MSM and to enhance health education for them. In addition, according to the results of our analysis, there are ethnic differences in HIV testing. Many studies have explored the relationship between racial/ethnic differences and HIV-related health services [49, 50]. However, few studies have been conducted in China. Based on the significant results of our study, it suggests the need to reduce racial/ethnic disparities in HIV testing and counselling services in future HIV interventions.

Our study has the following advantages. (1) This study is based on a large sample of MSM in Western China (N = 3184). The two phases of the HIV health services survey from 2013 to 2021 ensured continuity and robustness of the results. In addition, as a large sample of health service survey, the results can be applied in Western China. If it can perform well, it may provide new ideas for the national health service strategy. (2) MSM is a special population that is easily neglected by health services. Studying this special group can improve the attention of management departments to them, and identify MSM with high-risk characteristics to give priority to HIV health services. (3) A widely used and relatively mature theoretical model (Andersen’s Behavioral Model) is applied to HIV testing and counselling services to better understand and explain the factors that influence health services. It can also be applied to the health service survey of other special population, which has certain application value.

However, there are several problems as follows. (1) Our study used non-probability sampling, which may lead to bias in the results. But the MSM population is a marginal population that is difficult to reach, and we had to use such a sampling method. Similarly, many studies of factors influencing HIV testing in MSM population have encountered the same problem [32, 33]. (2) As a sensitive group, MSM pay great attention to privacy issues. As our survey was conducted in the form of face-to-face questionnaire, some sensitive issues (such as sexual behavior or sexual role) may be concealed. In the future, more attention should be paid to privacy protection of sensitive groups. (3) Our study integrates the results of 2 phases of the survey. In both surveys, we worked with different NGOs to recruit MSM population in different cities in Western China, trying to avoid participating multiple times. Also, the inclusion of time phase factor in the model for correction in the analysis minimizes the potential impact on the results. (4) Our study only involved a cross-sectional survey, which limited causal inference. Cohort studies can confirm these findings in subsequent studies.

## Conclusion

Our study explored the factors affecting HIV testing and counselling services based on Andersen’s Behavioral Model. HIV testing is relatively good in the MSM population in Western China, but HIV counselling is relatively low and there is a mismatch. Age and ethnicity in the predisposing factor, household register location and income in the enabling factor are variables that influence HIV health service utilization in MSM population. Need factor is the main factor determining the utilization of HIV testing and counselling services. MSM with low level of HIV knowledge and diagnosed with STD are the key population that needs to focus on. The government and relevant departments should strengthen the popularization of disease knowledge and the diagnosis and treatment of individual physical diseases. MSM population with high-risk characteristics should be identified as a priority in the future public health service delivery strategy. We hope that more studies will focus on the health services of this population in the future, investigate more needs and utilization, and provide new directions and ideas for AIDS prevention and policy making.

## Declarations

### Availability of data and materials

The datasets analyzed during the current study are available from the corresponding author on reasonable request.
